# The efficacy of molecular subtyping in predicting postoperative recurrence in breast-conserving therapy: a 15-study meta-analysis

**DOI:** 10.1186/1477-7819-12-212

**Published:** 2014-07-15

**Authors:** Jing Chen, Peng Jiang, Han-jin Wang, Jia-yi Zhang, Yang Xu, Mu-hong Guo, Bin Zhang, Chong-yin Tang, Hong-yong Cao, Shui Wang

**Affiliations:** 1 Department of General Surgery of Breast and Thyroid, Nanjing Hospital Affiliated to Nanjing Medical University, Nanjing, China; 2 Department of Urology, First Affiliated Hospital of Nanjing Medical University, Nanjing, China; 3 Department of General Surgery, Nanjing Hospital Affiliated to Nanjing Medical University, Nanjing, China; 4 Department of Breast Surgery, First Affiliated Hospital of Nanjing Medical University, Nanjing, China

**Keywords:** Breast cancer, Molecular subtypes, Breast-conserving therapy, Recurrence, Meta-analysis

## Abstract

**Background:**

Recent research displays that breast cancer (BC) is a heterogeneous disease and distinct molecular subtypes yield different prognostic outcomes.

**Methods:**

We conducted a meta-analysis to clarify the role of molecular subtypes in recurrence risk after breast-conserving therapy (BCT). Eligible studies of single- (ER, PR, Her-2, and p53) and triple-molecular (Luminal A, Luminal B, Her-2, triple-negative) subtypes were identified through multiple search strategies. Pooled hazard ratios with 95% confidence intervals were calculated to assess this research topic.

**Results:**

Fifteen studies involving 21,645 participants were included in the meta-analysis. Her-2 positive patients had a significantly higher recurrence risk in both overall merge (HR = 1.97, 95% CI: 1.41-2.75) and subtotal merge of local recurrence (LR) (HR = 1.93, 95% CI: 1.34-2.78). Significantly higher risk of recurrence was also observed in p53 positive patients by overall merge (HR = 1.78, 95% CI: 1.49 -2.12) and subtotal merge of LR (HR = 1.73, 95% CI: 1.44-2.07). When setting Luminal A as a baseline, Luminal B, Her-2, and triple-negative all showed significantly increased risk for both LR and distant recurrence (DR). Comparing triple-negative and non-triple-negative subtypes showed the biggest risk for overall recurrence (HR = 3.19, 95% CI: 1.91-5.31) and LR (HR = 3.31, 95% CI: 1.69-6.45).

**Conclusions:**

Our meta-analysis showed significant differences in recurrence risk among various molecular subtypes after BCT. Although Her-2 and p53 positive subtypes can be considered independent prognostic biomarkers for indicating high LR risk, triple-molecular biomarkers showed higher clinical value. Triple-negative subtype showed the highest recurrence risk among all subtypes, and adjuvant chemotherapy should be considered for it.

## Background

Breast-conserving therapy (BCT) is considered the standard treatment for early-stage breast cancer (BC) [1-3]. Though patients who undergo breast-conserving surgery (BCS) have a better quality of life and equivalent survival compared with those undergoing mastectomy [3], many randomized trials consistently demonstrate a measurable increased risk of local recurrence (LR) after BCS when compared to mastectomy [4-6], estimated at 1% per year [3,7-10].

The factors affecting recurrence are complex, including clinical and histological characteristics, with or without postoperative adjuvant radiotherapy and systemic therapy (chemotherapy and/or hormone therapy) [11]. Recent researches display that BC is a heterogeneous disease [12] and distinct molecular subtypes yield different prognostic outcomes [13-19]. These molecular markers mainly include estrogen receptor (ER), progesterone receptor (PR), human epidermal growth factor receptor 2 (HER-2), p53, and Ki67 [14,20]; these have immensely contributed to the selection of the optimal strategy for BCT [21-23]. However, the impact of molecular subtypes on LR or distant recurrence (DR) has not been systemically evaluated. Therefore, we conducted a meta-analysis to clarify the role of molecular subtypes in BC recurrence after BCT.

## Methods

### Search strategy

Original articles analyzing the hazard ratio (HR) of recurrence after BCT in different BC molecular subtypes were searched by online databases PubMed, Embase, and Web of Science. We selected studies carefully by the following sets of key words variably combined: ‘breast cancer’ , ‘breast-conserving surgery’ , ‘breast-conserving therapy’ , ‘recurrence’ , ‘hazard ratio’ , ‘molecular marker’ , and ‘molecular subtype’; the last search update was performed on 10 January 2014. All eligible studies published in English were reviewed, and their bibliographies were also examined for other relevant publications. Relevant review articles were manually searched to find additional eligible studies. If more than one article was published using the same series of study subjects, we only chose the latest or most complete study for this meta-analysis. All the studies enrolled in this meta-analysis have been performed with the approval of an appropriate ethics committee. Researches carried out on humans are all in compliance with the Helsinki Declaration.

### Inclusion and exclusion criteria

**Figure 1:**
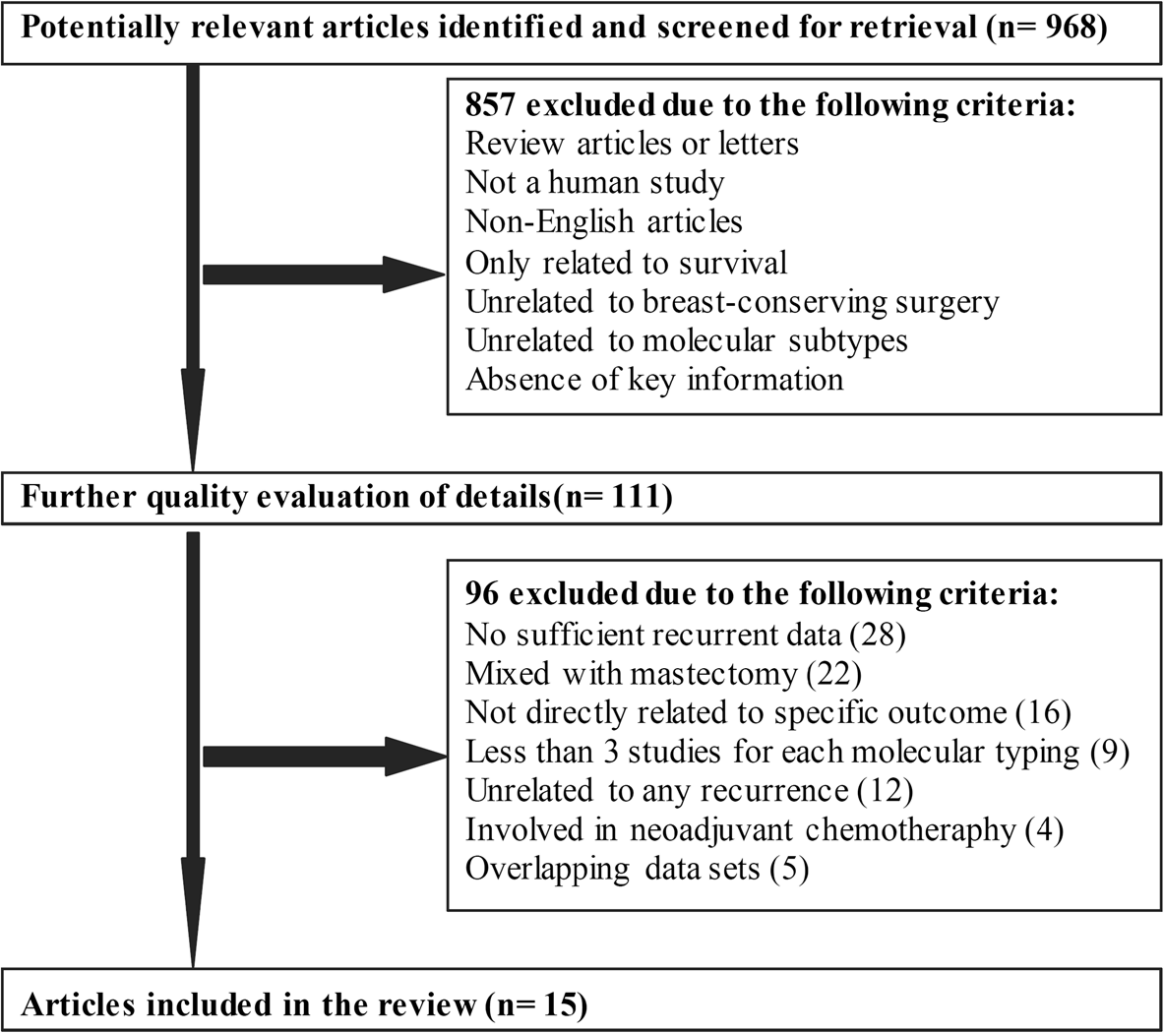
Flow diagram of study selection process.

We followed the guidelines of the critical checklist proposed by the Dutch Cochrane Centre Meta-analysis of Observational Studies in Epidemiology (MOOSE) [24]. Articles were identified as eligible when they fit the following criteria: (1) they performed BCS on BC patients; (2) they focused on postoperative recurrence; (3) they investigated the association between recurrence and different BC molecular subtypes. Altogether, 953 studies were excluded by exclusion criteria and further quality evaluation that are presented in Figure 1.

### Data extraction

All data were carefully extracted from eligible publications in duplicate by two co-authors (PJ and JZ). Any disagreements were resolved by discussion between the two authors. The extracted data elements in Table 1 include the followings: first author’s last name, publication year, case nationality, dominant ethnicity, study design, number of cases, median time to follow-up, percentage of patients who received adjuvant radiotherapy (RT), percentage of patients who received adjuvant systemic therapy (AST), types of recurrence, source of HR, and characteristics of enrolled cases.

**Table 1:** Main characteristics of the studies enrolled in the meta-analysis

**First author and publishing year**	**Case nationality**	**Dominant ethnicity**	**Study design**	**Case number**	**Follow-up median (m)**	**RT (%)**	**AST (%)**		**Types of recurrence**	**Source of HR**	**Characteristics of enrolled cases**
							**HT**	**CT**			
Hattangadi [30]	USA	Caucasian	R	1,223	70	100	77.0	46.0	TR	Reported	IBC, pT1-2 N0-3
Zauls [31]	USA	Caucasian	P	459	45	100	Total 62.1		LF	DE	DCIS or IBC, tumor size ≤3 cm, positive LNs ≤3
Han [32]	Canada	Caucasian	R	180	104.4	30.6	NM	NM	LR	Reported	DCIS with or without microinvasion
Millar [33]	Australia	Caucasian	P	498	64	100	49.0	38.0	LRR/IBTR/DM	DE	IBC
Moran [34]	USA	Caucasian	R	368	78	100	49.0	36.0	LR	DE	IBC, stage I-II, LN (+), margin (-)
Wong [35]	Singapore	Asian	R	541	72	100	68.0	36.0	LR/DM	Reported	IBC, LN (-)
Kim [36]	Korea	Asian	P	1,589	61	100	71.8	66.4	IBTR	Reported	IBC, tumor size ≤5 cm, age >40 years
Bantema [37]	Netherlands	Caucasian	R	752	41	100	38.7	35.4	LR/DM	Reported	IBC, stages I-III
Arvold [38]	USA	Caucasian	R	1,434	85	100	Total 91.0		LR	Reported	IBC, stage I-II
Truong [39]	Canada	Caucasian	R	5,688	71.7	100	36.6	26.7	LR/RR/LRR	Reported	IBC, pT1-2, positive LNs ≤3, M0
Sharon [40]	Canada	Caucasian	R	133	107	0.0	NM	NM	LR	Reported	DCIS, margin (-)
Roos [41]	Netherlands	Caucasian	R	39	39	51.3	0.0	NM	LR	DE	DCIS
Yau [42]	China	Asian	R	605	64.8	100	74.0	45.0	IBTR/DF	Reported	IBC, T1-2
Smith [43]	USA	Caucasian	R	8,724	60	73.0	NM	3.0	SIBC	Reported	IBC, age ≥70 years, tumor size ≤2 cm, LN (-)
Silvestrini [44]	Spain	Caucasian	R	635	72	78.1	0.0	0.0	LR	Reported	Resectable breast cancer, N0, M0

Study design is described as either prospective (P) or retrospective (R).

Radiotherapy (RT) is defined as accelerated partial breast irradiation (APBI), whole breast irradiation (WBI) or MammoSite brachytherapy (MB).

Adjuvant systemic therapy (AST) is defined as hormone/endocrine therapy (HT) only, chemotherapy (CT) only, or both.

*Abbreviations:m* month *HR* hazard ratio, *NM* not mentioned, *TR* true recurrence, *LF* local failure, *LR* local recurrence or relapse, *LRR* locoregional recurrence; *IBTR* ipsilateral breast tumor recurrence, *DM* distant metastasis, *RR* regional recurrence, *DF* distant failure, *SIBC* second ipsilateral breast cancer, *DE* dataextrapolated *IBC* invasive breast cancer, *DCIS* ductal carcinoma *in situ*, *LN* lymph node.

The extracted data elements in Table 2 show the HRs and 95% confidence intervals (CIs) of different recurrences among molecular subtypes. If HR was not reported directly, data were extracted from Kaplan-Meier curves of survival outcomes to extrapolate required data using the previously described methods [25-27]. We also wrote emails to the corresponding authors of enrolled studies to obtain additional information and original data needed for the meta-analysis.

**Table 2:** HRs and 95% CIs of various comparisons between molecular subtypes of breast cancer stratified by recurrence types

**First author and publishing year**	**Types of recurrence**	**LB** ***vs.*** **LA**	**Her-2** ***vs.*** **LA**	**TN** ***vs.*** **LA**	**TN** ***vs.*** **non-TN**	**ER -*vs.*** **+**	**PR -*vs.*** **+**	**Her-2 +** ***vs.*-**	**p53 +** ***vs.*-**
		**HR (95% CI)**	**HR (95% CI)**	**HR (95% CI)**	**HR (95% CI)**	**HR (95% CI)**	**HR (95% CI)**	**HR (95% CI)**	**HR (95% CI)**
Hattangadi [30]	TR	NM	NM	NM	4.80 (1.40, 15.80)^M^	NM	NM	NM	NM
Zauls [31]	LF	NM	NM	NM	NM	0.75 (0.15, 3.85)^U^	0.55 (0.12, 2.44)^U^	0.64 (0.07, 5.88)^U^	NM
Han [32]	LR	1.90 (0.90, 4.00)^U^	1.90 (0.90, 3.80)^U^	0.60 (0.10, 2.40)^U,DE^	NM	0.87 (0.48, 1.58)^U^	1.09 (0.61, 1.98)^U,DE^	1.98 (1.11, 3.53)^M,DE^	NM
Millar [33]	LR	2.48 (0.98, 6.29)^M^	1.93 (0.38, 9.75)^M^	3.94 (1.28, 12.11)^M^	NM	NM	NM	NM	1.20 (0.57, 2.55)^U,DE^
Millar [33]	DM	2.87 (1.33, 6.22)^M^	1.83 (0.39, 8.64)^M^	3.27 (1.14, 9.40)^M^	NM	NM	NM	NM	2.57 (1.30, 5.06)^U^
Millar [33]	LR + DM	2.71 (1.49, 4.90)^M,DE^	1.88 (0.61, 5.76)^M,DE^	3.89 (2.03, 7.44)^M,DE^	NM	NM	NM	NM	1.83 (1.10, 3.02)^U,DE^
Moran [34]	LR	NM	NM	NM	2.21 (0.63, 7.81)^M^	NM	NM	1.22 (0.30, 4.93)^M^	NM
Bantema [34]	LRR + DM	NM	NM	NM	3.03 (1.37, 6.67)^M^	NM	NM	NM	NM
Wong [35]	LR	NM	NM	NM	3.30 (1.20, 9.60)^U^	NM	NM	NM	NM
Wong [35]	DM	3.60 (1.10, 11.30)^U^	6.00 (1.60, 22.60)^U^	4.20 (1.10, 16.00)^U^	NM	NM	NM	NM	NM
Wong [35]	LR + DM	2.16 (0.85, 5.50)^M^	2.22 (1.08, 9.84)^M^	3.48 (1.22, 9.93)^M^	NM	NM	NM	NM	NM
Kim [36]	IBTR	1.55 (0.32, 7.51)^M^	0.55 (0.05, 6.34)^M^	1.17 (0.22, 6.26)^M^	NM	NM	NM	NM	NM
Arvold [38]	LR	2.10 (0.95, 4.80)^M^	5.20 (1.80, 15.00)^M^	3.90 (1.70, 9.00)^M^	NM	NM	NM	NM	NM
Truong [39]	LRR	NM	NM	NM	NM	1.29 (0.99, 1.69)^M^	NM	NM	NM
Sharon [40]	LR	NM	NM	NM	NM	NM	NM	1.93 (1.02, 3.65)^M^	NM
Roos [41]	LR	NM	NM	NM	NM	2.50 (0.42, 10.00)^U,DE^	1.11 (0.24, 5.00)^U,DE^	3.90 (0.80, 20.10)^U^	4.00 (0.90, 18.10)^U^
Yau [42]	IBTR	NM	NM	NM	NM	NM	NM	2.19 (0.76, 6.35)^U^	NM
Yau [42]	DF	NM	NM	NM	NM	NM	NM	2.17 (0.99, 4.75)^U^	NM
Yau [42]	IBTR + DF	NM	NM	NM	NM	NM	NM	1.57 (1.26, 1.97)^U,DE^	NM
Smith [43]	SIBC	NM	NM	NM	NM	NM	1.49 (1.00, 2.22)^U^	NM	NM
Silvestrini [44]	LR	NM	NM	NM	NM	1.12 (0.42, 2.97)^U,DE^	NM	NM	1.75 (1.44, 2.11)^U,DE^

The source of HRs and 95% CIs is derived from univariate analysis (^U^), multivariate analysis (^M^) or data-extrapolated (^DE^).

CI, confidence interval; DE, data-extrapolated; DF, distant failure; DM, distant metastasis; ER, estrogen receptor; Her-2, human epidermal growth factor receptor 2; HR, hazard ratio; IBTR, ipsilateral breast tumor recurrence; LA, Luminal A; LB, Luminal B; LF, local failure; LR, local recurrence or relapse; LRR, locoregional recurrence; NM, not mentioned; PR, progesterone receptor; SIBC, second ipsilateral breast cancer; TN, triple-negative; TR, true recurrence.

### Statistical analysis

All statistical analyses were conducted using Stata (version 11.0; StataCorp LP, College Station, TX, USA) and Excel (version 2007; Microsoft Corp., WA, USA). The aggregation of HRs and 95% CIs were calculated following Tierney’s method [27]. Forrest plots were used to estimate the effect of different molecular subtypes on recurrence after BCT. The heterogeneity assumption of pooled HRs was verified by Cochran’s Q-test, and the percentage of Higgins’ I-squared statistic (I^2^) was used to quantify the extent of heterogeneity explained by these characteristics of enrolled studies. If significant heterogeneity was observed (*P* <0.1 or I^2^ >50%), a random-effects model (Der Simonian- Laird method) was applied; otherwise, the fixed-effects model (Mantel- Haenszel method) was adopted [28]. Potential publication bias was determined by Egger’s linear regression test with a funnel plot [29].

To avoid the influence of heterogeneity among these studies, we also conducted a subgroup analysis stratified by different recurrence categories (LR and DR). We defined LR to include true recurrence, local failure, local recurrence or relapse, ipsilateral breast tumor recurrence, locoregional recurrence, and second ipsilateral BC. DR was defined as distant metastasis or failure, and recurrence was defined as any of LR and/or DR, the merger of LR and DR in the same study or not definitely mentioned. All *P* values were two-sided and a *P* value less than 0.05 was considered to be statistically significant.

## Results

### Summary of included studies

Fifteen studies involving 21,645 participants finally met the inclusion criteria [30-44]. The main features of eligible studies are summarized in Table 1. These studies collect data from the United States, Canada, Australia, Singapore, Korea, the Netherlands, China, and Spain. The dominant ethnicity of 13 enrolled studies is Caucasian [30-34,37-41,43,44], with only three studies executed in Asians [35,36,42]. Most enrolled studies are retrospective in design [30,32,34,35,37-44], except three prospective studies [31,33,36]. The median time to follow-up is in the range of 39 to 107 months.

A total of 20,890 patients (88.3%) received postoperative radiotherapy (RT), including accelerated partial breast irradiation, whole breast irradiation, or MammoSite brachytherapy. Adjuvant systemic therapy (AST) after BCS are defined as hormone/endocrine therapy (HT) only, chemotherapy (CT) only, or both. The directly reported rates range from 0.0% to 77.0% for HT and 0.0% to 66.4% for CT. Excluding studies that did not mention the respective rates of HT or CT, 6,288 patients (49.4%) underwent HT and 4,817 patients (22.5%) accepted CT (Table 1).

### Single-molecular subtypes and postoperative recurrence

Single-molecular subtypes are defined as the dichotomous status of a single receptor or protein, which will be excluded from the meta-analysis if less than three studies are found involved in. Altogether, four proteins (ER, PR, Her-2, and p53) are included in analyses.

**Figure 2:**
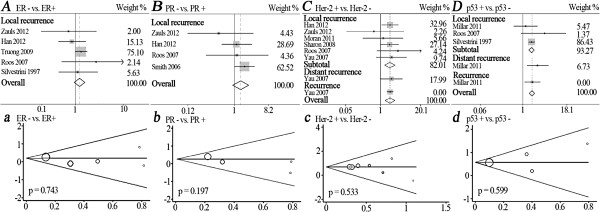
**Forest plots for recurrence risk of single-molecular typing after BCT in the following comparisons: ER - vs. ER+ (A), PR - vs. PR+ (B), Her-2+ vs. Her-2 - (C) and p53+ vs. p53- (D).** Squares and horizontal lines correspond to the study-specific HRs and 95% CIs, respectively. The area of the squares correlates the weight and the diamonds represent the summary HRs and 95% CIs. Begg's funnel plots for publication bias for the following comparisons: ER - vs. ER+ **(a)**, PR - vs. PR+ **(b)**, Her-2+ vs. Her-2 - **(c)** and p53+ vs. p53- **(d)**. ER, estrogen receptor; PR, progesterone receptor; Her-2, human epidermal growth factor receptor 2.

Six articles involved Her-2 typing [31,32,34,40-42] (Figure 2C) and we found that Her-2 positive patients had a significantly higher recurrence risk when compared to Her-2 negative individuals in both overall merge(HR = 1.97, 95% CI: 1.41-2.75) and LR subtotal merge (HR = 1.93, 95% CI: 1.34-2.78). A significantly higher risk of postoperative recurrence is also observed in p53 positive patients by overall merge (HR = 1.78, 95% CI: 1.49 -2.12) and LR subtotal merge (HR = 1.73, 95% CI: 1.44-2.07) in three studies [33,41,44] (Figure 2D). Five studies focus on ER typing [31,32,39,41,44], and no significant correlation is found between ER typing and LR (HR = 1.21, 95% CI: 0.96-1.53) (Figure 2A). Similarly, four eligible studies involving PR typing [31,32,41,43] did not show a significant result (HR = 1.29, 95% CI: 0.94-1.76) (Figure 2B). Above pooled HRs and 95% CIs are calculated by the fixed-effects model (Table 3).

**Table 3:** **Pooled HRs, 95% Cis, and** ***P*** **values of different dichotomous status of single receptor or protein stratified by recurrence types**

**Types of recurrence**	**ER -*vs.*** **+**			**PR -*vs.*** **+**			**Her-2 +** ***vs.*-**			**p53 +** ***vs.*-**		
	**N**	**HR (95% CI)**	***P***	**N**	**HR (95% CI)**	***P***	**N**	**HR (95% CI)**	***P***	**N**	**HR (95% CI)**	***P***
Overall	5	1.21 (0.96, 1.53)^a^	0.107	4	1.29 (0.94, 1.76)^a^	0.118	7	1.97 (1.41, 2.75)^a^	<0.01	4	1.78 (1.49, 2.12)^a^	<0.01
Local recurrence	5	1.21 (0.96, 1.53)^a^	0.107	4	1.29 (0.94, 1.76)^a^	0.118	6	1.93 (1.34, 2.78)^a^	<0.01	3	1.73 (1.44, 2.07)^a^	<0.01
Distant recurrence	0	-	-	0	-	-	1	2.17 (0.99, 4.75)	-	1	2.57 (1.30, 5.06)	-
Recurrence	0	-	-	0	-	-	1	1.57 (1.26, 1.97)	-	1	1.83 (1.10, 3.02)	-

Local recurrence is defined to include true recurrence (TR), local failure (LF), local recurrence or relapse (LR), ipsilateral breast tumor recurrence (IBTR), locoregional recurrence (LRR), or second ipsilateral breast cancer (SIBC). Distant recurrence is defined to include distant metastasis (DM) or distant failure (DF). Recurrence is defined to include any local and/or distant recurrence, which is the merge of LR and DR in the same study or not definitely mentioned.

^a^The HRs and 95% CIs of enrolled studies are pooled by the fixed-effects model.

CI, confidence interval; ER, estrogen receptor; Her-2, human epidermal growth factor receptor 2; HR, hazard ratio; N, number of studies; PR, progesterone receptor.

### Triple-molecular subtypes and postoperative recurrence

Triple-molecular subtypes are defined as the combination of dichotomous status of three receptors (ER, PR, and Her-2), including Luminal A (ER + and/or PR + and Her-2-), Luminal B (ER + and/or PR + and Her-2+), Her-2 (ER- and PR- and Her-2+), and triple-negative (ER- and PR- and Her-2-) [45].

**Figure 3:**
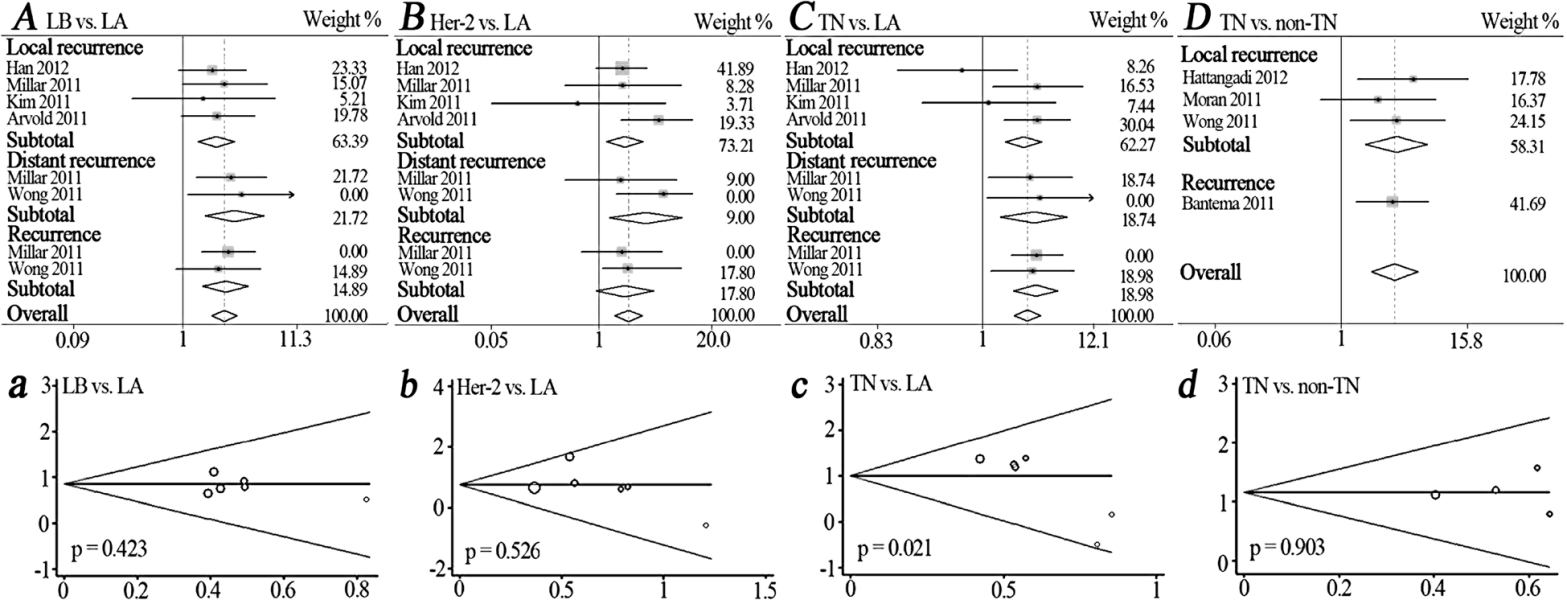
**Forest plots for the recurrence risk of triple-molecular typing after BCT in the following comparisons: LB vs. LA (A), Her-2 vs. LA (B), TN vs. LA (C) and TN vs. non-TN (D).** Squares and horizontal lines correspond to the study-specific HRs and 95% CIs, respectively. The area of the squares correlates the weight and the diamonds represent the summary HRs and 95% CIs. Begg's funnel plots for publication bias for the following comparisons: LB vs. LA **(a)**, Her-2 vs. LA **(b)**, TN vs. LA **(c)** and TN vs. non-TN **(d)**. LB, Luminal B; LA, Luminal A; Her-2, human epidermal growth factor receptor 2; TN, triple-negative.

By setting Luminal A as a baseline, the recurrence risk of patients with Luminal B, Her-2, or triple-negative subtypes are compared by overall and subtotal merge of five studies [32,33,35,36,38] (Table 2). We found that individuals with Luminal B had a significantly higher risk for total recurrence (HR = 2.23, 95% CI: 1.55 -3.19), LR (HR = 2.05, 95% CI: 1.31-3.23) or DR (HR = 3.08, 95% CI: 1.62-5.86) (Figure 3A). This significantly higher risk is also observed in patients with Her-2 subtype by overall merge (HR = 2.26, 95% CI: 1.42-3.60), LR subtotal merge (HR = 2.33, 95% CI: 1.35-4.02), and DR subtotal merge (HR = 3.64, 95% CI: 1.33-9.97) (Figure 3B). Triple-negative individuals also had significantly higher risk of overall recurrence (HR = 2.90, 95% CI: 1.84 -4.58), LR (HR = 2.64, 95% CI: 1.48-4.71), and DR (HR = 3.60, 95% CI: 1.57-8.25) (Figure 3C). Four studies focus on the comparison between triple-negative and non-triple-negative subtypes [30,34,35,37], and a significantly higher recurrence risk is observed in patients with triple-negative subtype by overall merge (HR = 3.19, 95% CI: 1.91-5.31) and LR subtotal merge (HR = 3.31, 95% CI: 1.69-6.45) (Figure 3D). Above pooled HRs and 95% CIs are calculated by the fixed-effects model (Table 4).

**Table 4:** **Pooled HRs, 95% CIs, and** ***P*** **values of different dichotomous status of ER, PR, and Her-2 protein stratified by recurrence types**

**Types of recurrence**	**LB** ***vs.*** **LA**			**Her-2** ***vs.*** **LA**			**TN** ***vs.*** **LA**			**TN** ***vs.*** **non-TN**		
	**N**	**HR (95% CI)**	***P***	**N**	**HR (95% CI)**	***P***	**N**	**HR (95% CI)**	***P***	**N**	**HR (95% CI)**	***P***
Overall	6	2.23 (1.55, 3.19)^a^	<0.01	6	2.26 (1.42, 3.60)^a^	0.001	6	2.90 (1.84, 4.58)^a^	<0.01	4	3.19 (1.91, 5.31)^a^	<0.01
Local recurrence	4	2.05 (1.31, 3.23)^a^	0.002	4	2.33 (1.35, 4.02)^a^	0.002	4	2.64 (1.48, 4.71)^a^	0.001	3	3.31 (1.69, 6.45)^a^	<0.01
Distant recurrence	2	3.08 (1.62, 5.86)^a^	0.001	2	3.64 (1.33, 9.97)^a^	0.012	2	3.60 (1.57, 8.25)^a^	0.002	0	-	-
Recurrence	2	2.54 (1.54, 4.19)^a^	<0.01	2	2.04 (0.93, 4.49)^a^	0.075	2	3.77 (2.17, 6.55)^a^	<0.01	1	3.03 (1.37, 6.67)	-

Local recurrence is defined to include true recurrence (TR), local failure (LF), local recurrence or relapse (LR), ipsilateral breast tumor recurrence (IBTR), locoregional recurrence (LRR), or second ipsilateral breast cancer (SIBC). Distant recurrence is defined to include distant metastasis (DM) or distant failure (DF). Recurrence is defined to include any local and/or distant recurrence, which is the merge of LR and DR in the same study or not definitely mentioned.

^a^The HRs and 95% CIs of enrolled studies are pooled by the fixed-effects model.

CI, confidence interval; Her-2, human epidermal growth factor receptor 2; HR, hazard ratio; LA, Luminal A; LB, Luminal B; N, number of studies; TN, triple-negative.

### Publication bias

Unexpectedly, the comparison of TN *vs.* LA (Figure 3c) shows obvious publication bias by Egger’s test (*P* = 0.021). However in the other seven molecular typing comparisons, the shapes of funnel plots seem symmetrical and the results of Egger’s test do not suggest any publication bias (all *P* >0.05) (Figure 2a, b, c, d and Figure 3a, b, d).

## Discussion

BC has become a major cause of morbidity and mortality in women [46]; there are an estimated 5.2 million BC survivors worldwide [47]. A recent study reported that the incidence of BC in the United States from 2000 to 2009 showed a recent increase, especially for early-stage disease (*in situ* and localized) in non-Hispanic blacks and Asian/Pacific Islanders [48]. In addition, from 1976 to 2009, the incidence of advanced BC rose significantly among young women in the US (from 1.53/100,000 to 2.9/100,000); this trend may be accelerating and seems confined to women aged 25 to 39 years [49].

Currently, local excision combined with adjuvant RT has been well-established as an optimal treatment strategy for early-stage BC [2,11,50,51]. This may be an overtreatment if RT is imposed on any patient after BCS. Recurrence risk in a considerable proportion of patients is sufficiently low, especially DCIS diagnosed through screening of healthy women [52]. In contrast, for patients with high recurrence risk where only RT may be insufficient, AST (HT and/or CT) should also be administered after BCS [30]. In our meta-analysis, 16 enrolled studies display extreme difference in rates for RT (0% to 100%), HT (0% to 77%), and CT (0% to 66.4%) based on the diversity of patient age, pathological types, clinical stages, and surgical margin (Table 1). Therefore, definite evaluation criteria are needed to identify patients at high recurrence risk from those at low risk, and to help choose a more precise postoperative treatment strategy for patients undergoing BCS.

Towards this goal, consideration must be given to the combination of underlying pathological and clinical characteristics. Recent studies have shown that molecular subtypes are prognostic for LR and DR after BCS, and immunohistochemical staining is often used to approximately identify these subtypes [18,19,53]. In this review, we take both single-molecular and triple-molecular typing into evaluation (Table 2). To our knowledge, this is the first meta-analysis study to comprehensively assess the effect of molecular subtypes on recurrence after BCT.

Our meta-analysis shows that there are significant differences in recurrence risk among various BC subtypes after BCT. First, we analyzed the efficacy of single-molecular subtypes. Although a slightly increased risk has been found in both ER negative and PR negative patients, there is no significant effect on LR after BCT. Only Her-2 positive patients display a significantly higher risk for local and overall recurrence when compared to Her-2 negative individuals (Table 3). It reveals that Her-2 positive status could be used clinically as an independent prognostic factor of high recurrence risk, and the status of Her-2 is the most important in the combined efficacy of triple-molecular subtypes. In addition, overexpression of p53 (p53 positive) is presumed to be a surrogate for TP53 mutations, which are associated with higher tumor grade [54]. Our results display that p53 positive patients have a significantly increased risk for overall recurrence and LR when compared to p53 negative (low expression) individuals (Table 3). Thus, p53 positive subtype should also be considered an important, independent prognostic biomarker for indicating high recurrence risk. P53 positive individuals are more necessary to accept adjuvant RT combined with or without AST and will benefit more than those with p53 negative BC for preventing postoperative recurrence because p53 positive subtype also predicts better responsiveness to both RT and AST [54]. Apart from the above mentioned four biomarkers, we did not find any other receptor or protein that met our inclusion criteria. Although Ki67 is generally accepted as one of the most important molecules for BC typing and has been studied over a long period of time [55], only one study was found focusing on our subject [33].

Recently, more studies have focused on the combined efficacy of ER, PR, and Her-2 receptors since the 12th St Gallen International Breast Cancer Conference (2011) Expert Panel adopted a new approach to BC classification for therapeutic purposes based on the recognition of intrinsic biological subtypes [55]. Therefore, we also put emphasis on the relationship between triple-molecular subtypes and recurrence risk after BCT. When compared with Luminal A, Luminal B, Her-2, and triple-negative subtypes all show significantly increased risk for both LR and DR. All HR values for overall recurrence based on triple-molecular typing are greater than 2.0, which can identify patients with higher recurrence risk and shows more clinical prediction value than analyses based on single-molecular typing (Table 4). For example, the HR value of LR (HR = 2.33) derived from triple-molecular comparison between Her-2 and Luminal A subtypes is larger than the HR value of LR (HR = 1.93) derived from single-molecular comparison between Her-2 positive and Her-2 negative subtypes. Therefore, we concluded that the clinical application of triple-molecular typing as a biomarker can better distinguish high-risk individuals compared to single-molecular typing.

Moreover, the comparison between triple-negative and non-triple-negative subtypes shows the biggest risk difference for overall recurrence (HR = 3.19) and LR (HR = 3.31) among all molecular typing comparisons. When setting Luminal A as a baseline, the HR value of triple-negative subtype (HR = 2.90) remains larger than that of Luminal B (HR = 2.23) or Her-2 subtypes (HR = 2.26) for overall recurrence (Table 4). Previous studies have shown that triple-negative receptor status is strongly associated with poor clinical outcomes [56], and that young women more frequently suffer from triple-negative tumors [57]. Thus, triple-negative subtype should be considered the biggest risk factor for recurrence and adjuvant CT should be administered in BCT.

Some limitations of this meta-analysis should be acknowledged. First, there are only three studies focused on Asians [35,36,42] and none on Africans in this meta-analysis, hindering comprehensive investigation of the association between BC molecular typing and recurrence risk after BCT. Second, the sample sizes of enrolled researches (from 39 to 8,724) vary widely, and therefore the statistical power or weight of each study is greatly different, inevitably causing bias to varying degrees. Third, the number of original studies focusing on this topic is insufficient, especially studies related to DR risk.

## Conclusions

Our meta-analysis represents a quantified synthesis of all published studies and shows significant differences in recurrence risk among various molecular subtypes after BCT. Her-2 positive and p53 positive subtypes can be considered independent prognostic biomarkers for indicating high LR risk, but triple-molecular biomarkers exhibit higher clinical value than single-molecular biomarkers. Moreover, triple-negative subtype shows the biggest risk for overall recurrence and LR among all molecular subtypes and adjuvant CT should be considered in BCT. Considering the insufficient number of original studies, further research with different ethnicities is needed on this topic, especially the intensity of association between molecular typing and DR risk after BCT.

## Abbreviations

AST: Adjuvant systemic therapy; BC: Breast cancer; BCS: Breast-conserving surgery; BCT: Breast-conserving therapy; CIs: Confidence intervals; CT: Chemotherapy; DR: Distant recurrence; ER: Estrogen receptor; HER-2: Human epidermal growth factor receptor 2; HR: Hazard ratio; HT: Hormone/endocrine therapy; I^2^: I-squared statistic; LR: Local recurrence; PR: Progesterone receptor; RT: Adjuvant radiotherapy.

## Competing interests

The authors declare that they have no conflict of interest.

## Authors’ contributions

JC, PJ, and SW conceived and designed the study. PJ, J-yZ, and YX collected the data. H-jW contributed to quality assessment. M-hG, BZ, and C-yT performed statistical analyses. JC and H-yC drafted and revised the manuscript. All authors read and approved the final version.
